# Potential Molecular Mimicry Proteins Responsive to α-pinene in *Bursaphelenchus xylophilus*

**DOI:** 10.3390/ijms21030982

**Published:** 2020-02-01

**Authors:** Fanli Meng, Yongxia Li, Zhenkai Liu, Xuan Wang, Yuqian Feng, Wei Zhang, Xingyao Zhang

**Affiliations:** 1 Laboratory of Forest Pathogen Integrated Biology, Research Institute of Forestry New Technology, Chinese Academy of Forestry, Beijing 100091, China; mfl@caf.ac.cn (F.M.); zhenkailiu@caf.ac.cn (Z.L.); xwang@caf.ac.cn (X.W.); fengyq@caf.ac.cn (Y.F.); zhangwei1@caf.ac.cn (W.Z.); xyzhang@caf.ac.cn (X.Z.); 2 Co-Innovation Center for Sustainable Forestry in Southern China, Nanjing Forestry University, Nanjing 210037, China

**Keywords:** pine wood nematode, molecular mimicry proteins, α-pinene

## Abstract

*Bursaphelenchus xylophilus* is a nematode species that has damaged pine trees worldwide, but its pathogenesis has not been fully characterized. α-pinene helps protect host species during the early *B. xylophilus* infection and colonization stages. In this study, we identified potential molecular mimicry proteins based on a comparative transcriptomic analysis of *B. xylophilus*. The expression levels of three genes encoding secreted *B. xylophilus* proteins were influenced by α-pinene. We cloned one gene encoding a thaumatin-like protein, *Bx-tlp-2* (accession number MK000287), and another gene encoding a cysteine proteinase inhibitor, *Bx-cpi* (accession number MK000288). Additionally, α-pinene appeared to induce *Bx-tlp-1* expression, but had the opposite effect on *Bx-cpi* expression. An analysis of the expression of the potential molecular mimicry proteins in *B. xylophilus* infecting pine trees revealed that the α-pinene content was consistent with the expression levels of *Bx-tlp-1* (*Bx-cpi*) and *Pm-tlp* (*Pm-cpi*) over time. Thus, these genes likely have important roles contributing to the infection of pine species by *B. xylophilus*. The results of this study may be relevant for future investigations of the functions of *Bx-tlp-1*, *Bx-tlp-2* and *Bx-cpi*, which may provide a point to explore the relationship between *B. xylophilus* and host pines.

## 1. Introduction

Pine wilt disease (PWD), due to the pine wood nematode (PWN) *Bursaphelenchus xylophilus* (Steiner & Buhrer) Nickle [1], has extensively damaged pine trees in Asia (Japan, China and South Korea) [2,3,4] and Europe (Portugal and Spain) [5,6,7,8]. Since 1982, this disease has spread quickly and caused enormous losses in China [9]. Some *Pinus* species are the main hosts for *B. xylophilus* [10,11,12]. Although diverse methods have been used to study *B. xylophilus*, the mechanism underlying its pathogenicity has not been fully characterized.

Pine trees release terpenoids as a defense response to nematode invasions [13,14]. For example, α-pinene represents a large proportion of the monoterpenes produced by pines [15]. In healthy *Pinus massoniana*, the ratio of α-pinene to β-pinene is 1:0.1 [16,17,18,19]. Kuroda et al. [20] inoculated *Pinus thunbergii* seedlings with PWNs, and observed that monoterpene and sesquiterpene concentrations increased two to four times in the xylem soon after the PWN invasion.

The *B. xylophilus* genome was sequenced in 2011 [21]. Additionally, the molecular changes mediating the resistance of *B. xylophilus* to α-pinene have been examined via a comparative transcriptomic analysis of nematodes [22]. The results of this previous study, combined with the findings of other *B. xylophilus* investigations, including a large-scale proteomic analysis [23], may be relevant for future studies on PWD and the pathogenicity of PWNs.

Pathogenesis-related (PR) proteins are crucial for plant defenses against pathogens and abiotic stresses [24]. Thaumatin-like proteins (TLPs) have been widely studied in plants, fungi and animals [25]. An earlier investigation on the *B. xylophilus* secreted proteome revealed that two TLPs and one cysteine proteinase (CP) inhibitor are highly similar to the proteins in pine trees (i.e., molecular mimicry) [23]. Molecular mimicry has helped nematodes avoid the effects of plant defense responses [26]. Moreover, *Brugia malayi* secretes a protein similar to the migration inhibitory factor (MIF) [27,28], which may be functionally similar to human monocytes. *Globodera rostochiensis* secretes CLAVATA3/ESR (CLE), which may interact with plant CLE peptide ligands to inhibit plant development [29,30]. Additionally, some *Meloidogyne* spp. proteins are reportedly similar to host plant proteins [31]. Another study proved that a *B. xylophilus* TLP (Bx-TLP-1) is similar to *Pinus monticola* TLP-S3 (ADB97933.1), based on BLAST and structural analyses [32]. A *P. massoniana* TLP gene (*Pm-tlp*) was identified and used to study the expression of TLP-encoding genes in *B. xylophilus* and *P. massoniana* [33].

Cysteine proteinases, which are also known as thiol proteases because of the function of a core catalytic cysteine, mediate the hydrolysis of proteins [34]. Cysteine proteinases expressed in the esophageal gland and intestinal cells are the main “digestive enzymes” of nematodes [35]. Previous studies indicated that CPs are important for tissue and cellular invasion [36], nutrient acquisition related to embryogenesis [37], molting [38], host protein processing [39] and immunoevasion [40]. Because many animal and plant diseases are closely related to CPs, there has been increasing interest in research regarding their biological and enzymatic characteristics, including in *B. xylophilus*. For example, a *B. xylophilus* CP, Bx-CPL-1, is related to fecundity and growth, with an abnormal *Bx-cpl-1* gene, leading to decreased adult reproduction rates [41]. Additionally, *Bx-cpl* expression levels are the highest during the *B. xylophilus* egg stage. After the infection of a susceptible host, the *Bx-cpl* expression levels increased, peaking during the initial stage of PWD [42]. However, a CP inhibitor (cystatin) can tightly, but reversibly, bind to CPs [43]. Cystatins regulate normal physiological processes, with decreases in their abundance possibly leading to disease. They may also participate in defenses against microbial infections [44]. Cystatins are crucial for several processes under normal and diseased conditions, including intracellular protein degradation, and may be important for controlling antigen presentation. Furthermore, the increased activity of some cystatins may be essential for responses to several pathogenic parasites or bacteria.

Because the pinene content and water dynamics influence the cavitation of pines during *B. xylophilus* pathogenesis, investigating the relationship between specific genes and pinene is necessary. In this study, we identified potential molecular mimicry proteins based on comparative *B. xylophilus* transcriptomics data [22]. The *B. xylophilus Bx-tlp-2* and *Bx-cpi* genes were cloned to investigate the effects of α-pinene treatments on the expression of *Bx-tlp-1*, *Bx-tlp-2* and *Bx-cpi*. The results described herein may be useful for verifying and characterizing the interaction between *B. xylophilus* and host pines.

## 2. Results

### 2.1. Cloning of Bx-tlp-2 and Bx-cpi

**Figure 1:**
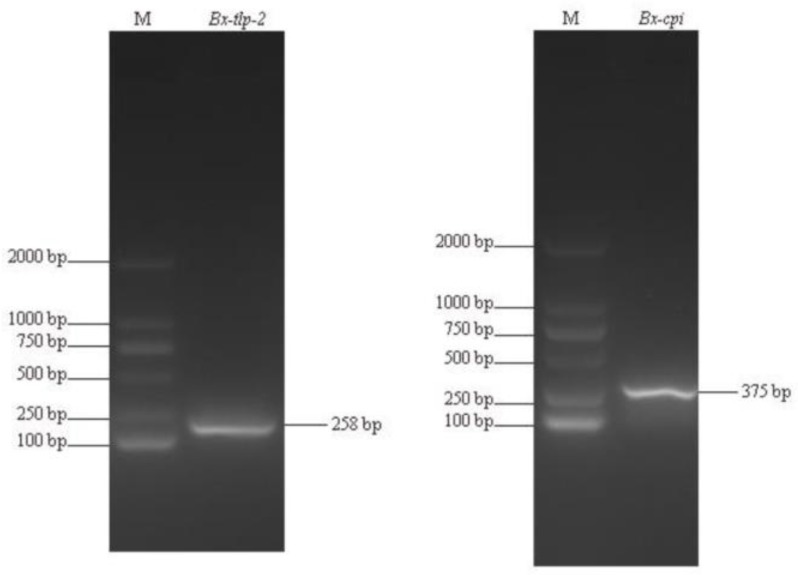
RT-PCR gel electrophoresis detection of *Bx-tlp-2* and *Bx-cpi.* M—DNA ladder.

The PCR-amplified target cDNA sequences were analyzed by 1% agarose gel electrophoresis. The *B. xylophilus* gene encoding thaumatin-like protein-2 (TLP-2) was 258 bp, whereas the CP inhibitor gene was 375 bp (Figure 1).

### 2.2. Analysis of the Bx-TLP-2 and Bx-CPI Proteins

**Figure 2:**
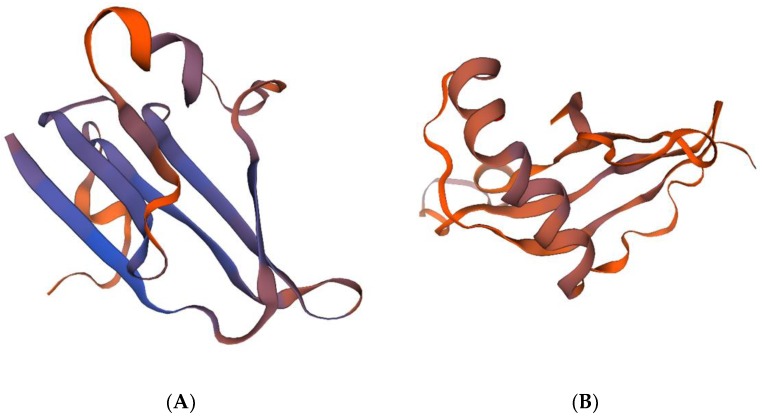
The picture of three-dimensional structure of (**A**) Bx-TLP-2 and (**B**) Bx-CPI.

**Figure 3:**
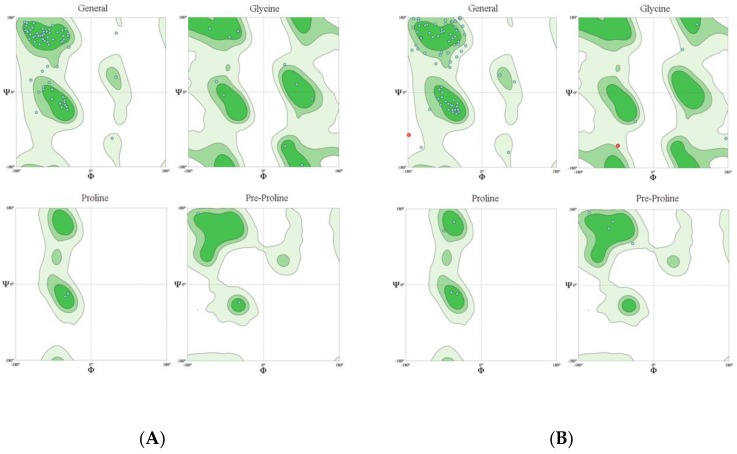
Evaluation of the three-dimensional structure by MolProbity: (**A**) Bx-TLP-2 and (**B**) Bx-CPI.

**Figure 4:**
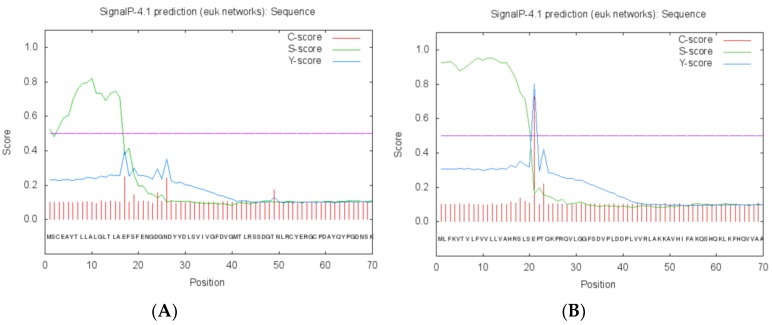
Signal peptide prediction analysis of (**A**) Bx-TLP-2 and (**B**) Bx-CPI.

The *Bx-tlp-2* cDNA fragment generated from *B. xylophilus* RNA encodes an 86-amino acid protein, which includes 12 acidic amino acids (Asp and Glu) and five basic amino acids (Arg and Lys; Table 1). The amplified *Bx-cpi* cDNA sequence encodes a 124-amino acid protein, which includes five acidic amino acids (Asp and Glu) and 22 basic amino acids (Arg and Lys; Table 2). The predicted protein structural features suggest that these proteins are hydrophilic and belong to the secretory protein family. Alpha-helices and beta-sheets form approximately 1.48% and 36.55% of Bx-TLP-2, respectively, whereas they represent about 40.18% and 27.75% of Bx-CPI, respectively. The MolProbity analysis was used to evaluate the quality of the three-dimensional structures of the proteins (Figure 2) and to show the percentage of amino acid residues present in favored, allowed and outlier regions. The residues falling in the most favored regions of the Ramchandran plot obtained by MolProbity amounted to 90.24% and 77.78% (>70%) for Bx-TLP-2 and Bx-CPI, respectively (Figure 3). This provided insight into the correctness of the modeled structures, and indicated the rationality of the protein structure prediction. Additionally, both proteins appeared to lack a transmembrane region. Amino acids 1–16 form the Bx-TLP-2 signal peptide, whereas the signal peptide of Bx-CPI comprises amino acids 1–20 (Figure 4). Moreover, the results revealed that Bx-TLP-2 is similar to a *Panicum hallii* TLP (XP_025808117.1), based on BLAST analysis. It shares a 52.05% identity with this protein, with over 85% query coverage. Additionally, the Bx-CPI protein is similar to the CP inhibitor five-like protein of *Helianthus annuus* (XP_022033148.1), which shares a 32.5% identity with this protein, with over 62% query coverage. Furthermore, Bx-CPI contains an N-terminal cathepsin propeptide inhibitor domain (I29), similar to Bx-CPL-1 and *P. massoniana* Pm-CPL (Figure S1).

### 2.3. Genetic Relationship between Bx-tlp-2 and Bx-cpi

**Figure 5:**
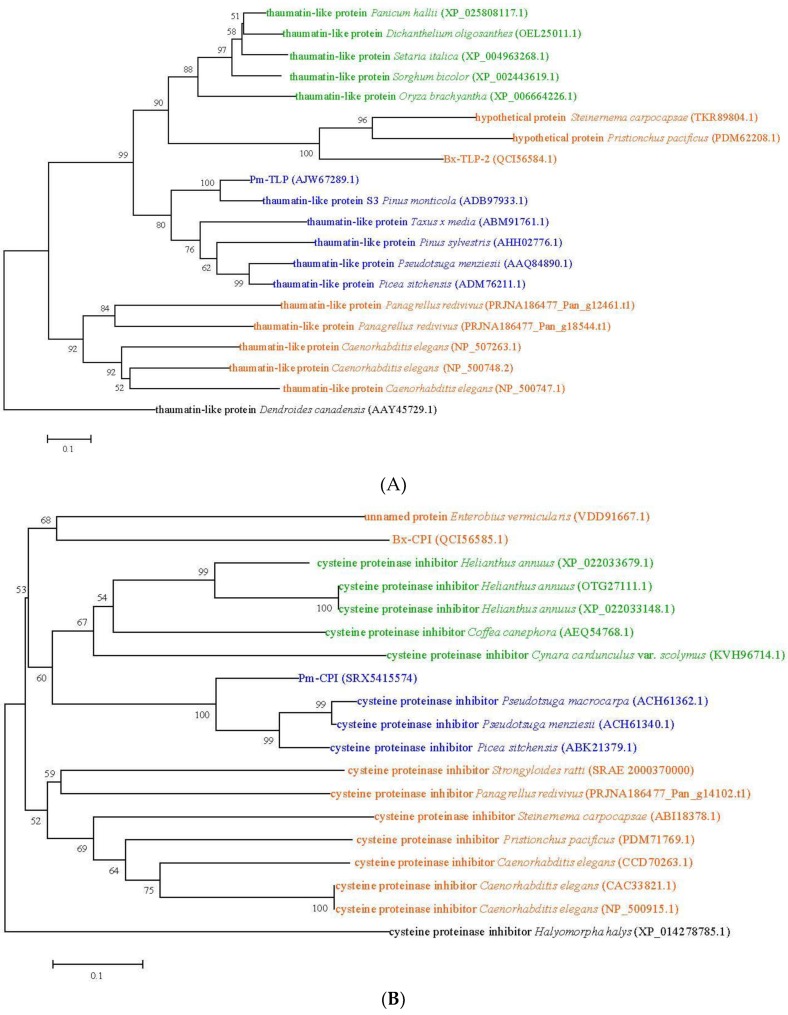
The phylogenetic tree of (**A**) Bx-TLP-2 and (**B**) Bx-CPI. The phylogenetic tree was reconstructed by MEGA-X software with the neighbor-joining (N-J) method, and the bootstrap value was set for 500 replicates. Species have identical colors: green indicates herbs, blue indicates woody plants, orange indicates nematodes and black indicates insects.

Of the two cloned cDNA sequences, one was *B. xylophilus Bx-tlp-2* (accession number MK000287), which encodes a TLP, and the other was *B. xylophilus Bx-cpi* (accession number MK000288), which encodes a CPI. A phylogenetic tree was constructed based on the alignment of the Bx-TLP-2 and Bx-CPI protein sequences, along with other protein sequences from herbs, woody plants and animals (nematode and insect). As shown in Figure 5A, the herb, woody plant and animal (nematode) TLP sequences formed three broad clades, and Bx-TLP-2 was similar to a *Steinernema carpocapsae* (Steinemematidae) hypothetical protein and a *Pristionchus pacificus* (Diplogasteridae) hypothetical protein, which was clustered in the broad plant clade. On the other hand, the *Panagrellus redivivus* (Panagrolaimidae) and *Caenorhabditis elegans* (Rhabditidae) TLPs were clustered in the broad nematode clade. Figure 5B showed that Bx-CPI was similar to an *Enterobius vermicularis* (Oxyuridae) unnamed protein, which was also not clustered in the broad nematode clade, while the *Strongyloides ratti* (Strongyloididae), *P. redivivus* (Panagrolaimidae), *S. carpocapsae* (Steinemematidae), *P. pacificus* (Diplogasteridae) and *C. elegans* (Rhabditidae) CPI sequences formed the broad nematode clade. Furthermore, the branch lengths shown in the tree were much longer for the *B. xylophilus* and *S. carpocapsae* (*E. vermicularis*) proteins, suggesting more molecular differences. The results revealed that there was a higher sequence similarity between the Bx-TLP-2 (Bx-CPI) protein sequence and the plant protein sequences, which indicated that they may have similar functions.

### 2.4. Expression of Bx-tlp-1, Bx-tlp-2, Bx-cpi and Bx-cpl-1

**Figure 6:**
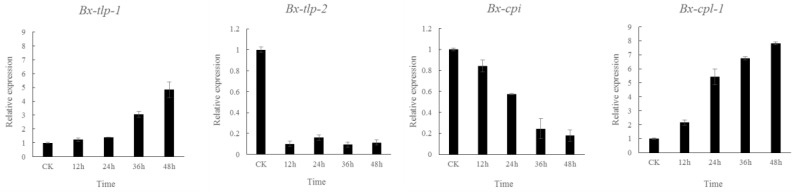
Gene expressions of *Bx-tlp-1*, *Bx-tlp-2*, *Bx-cpi* and *Bx-cpl-1*. CK—controls; 12 h—soaked for 12 h; 24 h—soaked for 24 h; 36 h—soaked for 36 h; 48 h—soaked for 48 h. There were three biological replications for each experiment. The error line means standard deviation of the mean.

The expression of the *B. xylophilus Bx-tlp-1* and *Bx-cpl-1* genes increased from 12 to 48 h after the PWNs were mixed with the α-pinene miscible solution. In contrast, the *Bx-cpi* expression level exhibited the opposite pattern over the same period. Additionally, the *Bx-tlp-2* expression was extremely low (Figure 6). Therefore, the quantitative real-time PCR (qPCR) results were consistent with the transcriptomic data, confirming the accuracy of our experiments.

### 2.5. Expression Patterns of Potential Molecular Mimicry Proteins from B. xylophilus and P. massoniana

**Figure 7:**
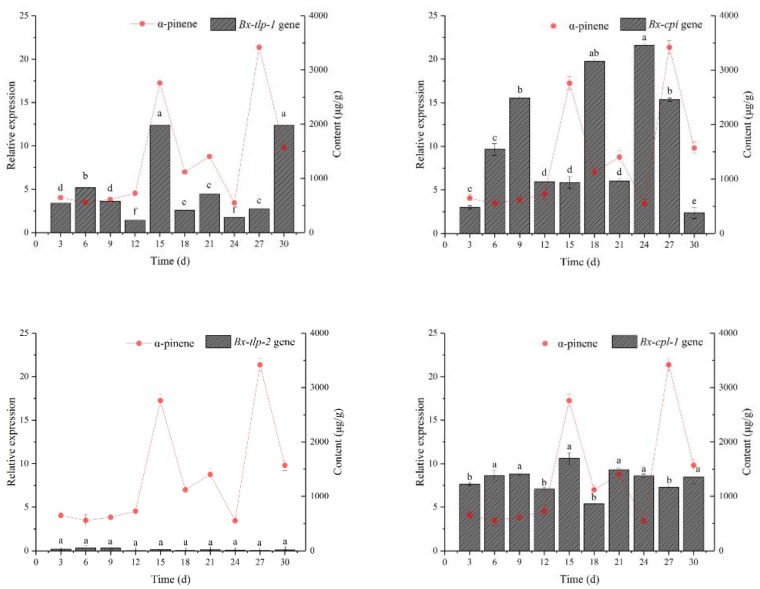

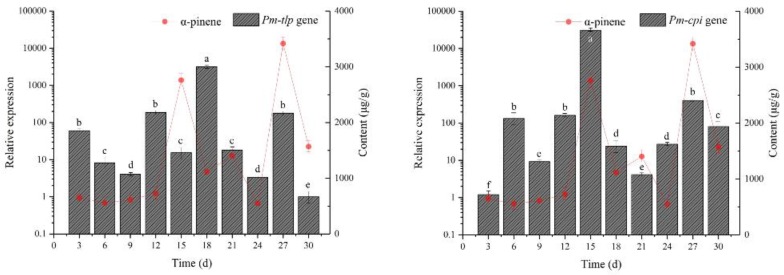
Expression analysis of the potential molecular mimicry proteins from *B. xylophilus* and *P. massoniana.* There were three biological replications for each experiment. The error line means standard deviation of the mean. Bars with different letters indicate significant differences among the treatments, as defined by Duncan’s test (*p* < 0.05).

The α-pinene content of *P. massoniana* inoculated with *B. xylophilus* was determined over time (Figure 7). The α-pinene level peaked at days 15 and 27. The *Bx-tlp-1* expression level increased on days 6, 15 and 30, whereas *Bx-tlp-2* was barely expressed throughout the analyzed period. The *Pm-tlp* expression level was upregulated on days 3, 12, 18 and 27. An increased *Bx-cpi* expression was detected at days 9, 18, 24 and 27. In contrast, there were no obvious *Bx-cpl-1* expression trends, whereas the *Pm-cpi* expression increased on days 6, 12, 15 and 27. The *Bx-tlp-1* and *Pm-tlp* expression trends were consistent with the changes in the α-pinene content (days 15 and 27). Moreover, the *Bx-tlp-1* expression level decreased as the *Pm-tlp* expression level increased. Furthermore the α-pinene content increased as the *Bx-cpi* expression level decreased. Decreases in the *Bx-cpi* expression were accompanied by increases in the *Pm-cpi* expression.

## 3. Discussion

Pinenes, which are the main secondary metabolites in needle-leaved species, help protect trees against pathogens [14]. Consequently, their abundance is a critical factor for evaluating the defenses of hosts against PWN invasion [15,45]. In this study, we identified potential molecular mimicry proteins based on the comparative transcriptomic analysis of *B. xylophilus* [22], which may mimic host plant defense systems [23]. We cloned the *B. xylophilus* genes *Bx-tlp-2* and *Bx-cpi* to investigate the expression of *Bx-tlp-1*, *Bx-tlp-2* and *Bx-cpi* in response to α-pinene treatments. The results indicated that the *Bx-tlp-1* expression was upregulated when the PWNs were combined with an α-pinene miscible solution, whereas the *Bx-cpi* expression was downregulated and the *Bx-tlp-2* expression was almost undetectable under the same conditions. The qPCR data were consistent with the results of the transcriptomic analysis (Table S1).

In PWNs, *Bx-tlp-1* encodes a TLP that is similar to *P. monticola* TLP-S3 (ADB97933.1) [32]. After PWNs infect *P. massoniana*, the *Bx-tlp-1* and *Pm-tlp* expression levels are affected by the changes in the pinene content of the host [33]. Increasing concentrations of volatile terpenes may induce the expression of *Bx-tlp-1*, with the encoded protein possibly mimicking plant proteins to disrupt the host defense system and secondary metabolism. In *B. xylophilus*, *Bx-tlp-2* encodes another TLP, but this gene was hardly ever expressed in the control and treated samples in this study. Specifically, the *Bx-tlp-2* reads per kilobase per million mapped reads (RPKM) value was ≤ 1, based on the transcriptomic analysis. Thus, whether this gene contributes to the PWN infestation of pine trees remains unclear. The *Bx-cpi* gene encodes a cystatin [23], which is a tight-binding reversible inhibitor of CPs [43]. Moreover, cystatins participate in defenses against microbial infections [44]. In *B. xylophilus*, Bx-CPL-1 reportedly influences reproduction and growth, with mutations to *Bx-cpl-1* adversely affecting the reproduction rates of adults [41]. In our study, we observed that the *Bx-cpi* expression level decreased in response to α-pinene, whereas the *Bx-cpl-1* expression was upregulated by the same treatment. Additionally, similar to *B. xylophilus* Bx-CPL-1 and *P. massoniana* Pm-CPL, the Bx-CPI protein includes a cathepsin propeptide inhibitor domain (I29). The I29 domains of Bx-CPL-1 and Pm-CPL are formed by amino acids 86–137 and 42–98, respectively. Additionally, I29 comprises an α-helical domain, which was across the substrate-binding site to prevent access. The enzyme was activated by removing this region with proteolytic cleavage [46]. Thus, we speculate that in response to pinene treatments, Bx-CPI may mediate *Bx-cpl-1* expression to promote PWN reproduction, which is consistent with the findings of a previous study [17].

In this investigation, we cloned *B. xylophilus* genes encoding thaumatin-like protein-2 (Bx-TLP-2) and a CP inhibitor (Bx-CPI). The *Bx-tlp-2* gene consists of 258 bp encoding an 86-amino acid protein with a molecular weight of 9.35 kDa. In contrast, *Bx-cpi* comprises 375 bp encoding a protein with 124 amino acids and a molecular weight of 13.99 kDa. Moreover, Bx-CPI can bind tightly to CP. MolProbity is a structure-validation software package that evaluates how reasonable a predicted structure is, relying on hydrogen placement within a structure. Further evaluation resulted in about 90.24% residue of Bx-TLP-2 and 77.78% residue of Bx-CPI falling in the favorable regions of the Ramchandran plot, which suggested that the prediction of three-dimensional structures (Bx-TLP-2 and Bx-CPI) was rational, based on the MolProbity analysis. A phylogenetic analysis revealed that there was a higher sequence similarity between the Bx-TLP-2 (Bx-CPI) protein sequence and the plant protein sequences, which indicated that they may have similar functions. The results provided the possibility for the Bx-TLP-2 and Bx-CPI to be molecular mimicry proteins or effectors. It was also reported that *B. xylophilus* TLP (Bx-TLP-1) may function as an effector molecule that can manipulate plant hosts [47]. The similarity of these secreted proteins to plant proteins suggests that they may mimic the components of the host plant defense systems, and may be attributed to the competitive co-evolution between nematodes and hosts, or a close ancestor of nematodes and its host.

An earlier study indicated that the genes encoding PR-5 (TLP) and PR-6 (CPI) are obviously upregulated in *P. thunbergii* seedlings during *B. xylophilus* infections [48]. The results of the current study revealed high *Pm-tlp* expression levels at days 3, 12 and 27, whereas *Bx-tlp-1* was highly expressed on days 6, 15 and 30. Additionally, the *Pm-cpi* expression level was relatively high on days 6, 15 and 27. In contrast, *Bx-cpi* was highly expressed on days 9, 18 and 24. These results imply that the upregulated expression of *Bx-tlp-1* (*Bx-cpi*) occurs after the induced expression of *Pm-tlp* (*Pm-cpi*) in infected pines, suggesting that the expression patterns of *Bx-tlp-1* (*Bx-cpi*) and *Pm-tlp* (*Pm-cpi*) may be related. These findings indicate that the three analyzed secreted *B. xylophilus* proteins may function as effectors to mediate the host defense responses [49], but whether they can mediate host reactions and enhance the pathogenicity of PWNs will need to be determined in future studies.

In conclusion, the results presented herein confirm that α-pinene affects the expression of three secreted *B. xylophilus* proteins that may mimic the host proteins involved in the plant defense systems. We speculated that increases in the volatile terpene concentrations may upregulate the expression of *Bx-tlp-1*, with the encoded protein mimicking the host pine tree proteins, thereby disrupting the defense system and secondary metabolism. The *Bx-cpi* expression level decreases in response to α-pinene, which may mediate the *Bx-cpl* expression to promote PWN reproduction. These effects contribute to the ability of *B. xylophilus* to infect susceptible hosts. Our study data may be useful for the future functional characterization of *Bx-tlp-1*, *Bx-tlp-2* and *Bx-cpi* regarding their roles during the *B. xylophilus* infections of host pine species, which may provide new insight relevant to the control of PWN and PWD.

## 4. Materials and Methods

### 4.1. Materials

The NXY61 strain of *B. xylophilus* was isolated from infested *P. massoniana* wood chips collected from Zhejiang, China, and then stored in the Forest Pathogen Integrated Biology Laboratory of the Chinese Academy of Forestry, Beijing, China. The three-year-old *P. massoniana* seedlings examined in this study were grown in a greenhouse set at 25 °C with 80% relative humidity.

### 4.2. RNA Isolation and cDNA Cloning

The TRIzol reagent (Thermo Fisher Scientific, Waltham, MA, USA) was used to extract the total RNA from *B. xylophilus.* A 1.0% agarose gel electrophoresis was used to evaluate the quality of RNA, and the purity of the RNA was evaluated by the ND-1000 spectrophotometer (NanoDrop Technologies, Wilmington, DE, USA). The RNA was used as a template to synthesize the cDNA with the PrimeScript™ RT reagent Kit using gDNA Eraser (TaKaRa, Kusatsu, Japan).

Reverse transcription PCR was performed with TaKaRa Taq™ DNA polymerase and primers designed to specifically amplify the *B. xylophilus Bx-tlp-2* and *Bx-cpi* fragments. The resulting amplicons were cloned into the pGM-T vector and sequenced at the Beijing Genomics Institute (Shenzhen, China) so as to ensure accuracy.

### 4.3. Verification of the Relationship between α-pinene and the Potential Molecular Mimicry Proteins in Bursaphelenchus xylophilus

A 137.6 mg/mL α-pinene solution [17] was comprised by serial dilution with ddH_2_O containing 0.5% (*w*/w) Triton X-100, as this may represent the α-pinene concentration in healthy pines or pines infested with *Monochamus alternatus* (i.e., PWN vector). The prepared solution and approximately 2500 PWNs (mixture of juveniles and adults) in 250 μL of water were combined in 1.5-mL tubes. The control samples comprised 0.5% (*w*/*w*) Triton X-100 prepared in distilled water and PWNs. The nematodes were maintained under the same conditions as those used for colony maintenance. The samples were collected at 12, 24, 36 and 48 h after initiating the experiment.

A qPCR assay was completed to analyze the expression of the genes encoding the potential molecular mimicry proteins (*Bx-tlp-1*, *Bx-tlp-2* and *Bx-cpi*). Specifically, the qPCR assay was performed in triplicate with TB Green^®^ Premix Ex Taq™ (TaKaRa, Kusatsu, Japan) and 480 II LightCycler system (Roche Diagnostics Ltd, Basel, Switzerland). The qPCR conditions followed one cycle of denaturation at 95 °C for 30 s, followed by 40 cycles at 95 °C for 5 s and 60 °C for 35 s. The β-actin gene of *B. xylophilus* was used as a reference control with the NCF/NCR primer pair (Table S2). The cycle threshold (ΔΔCt) method was used to calculate the gene expression levels. There were three biological replicates in the experiments.

### 4.4. Expression Analysis of the Potential Molecular Mimicry Proteins from Bursaphelenchus xylophilus and Pinus massoniana

Three-year-old *P. massoniana* seedlings were inoculated with 3000 nematodes (including juveniles and adults). The α-pinene content, as well as the *B. xylophilus* and *P. massoniana* gene expression levels, were measured as described by Meng [33]. The *P. massoniana* actin gene was amplified as a reference control with the ACF/ACR primer pair (Table S2). There were three biological replicates in the experiments.

### 4.5. Data Analysis

The DNAMAN 6.0 program (Lynnon Biosoft Bioinformatic Solutions, San Ramon, CA, USA) was used to translate the DNA sequences into amino acid sequences, after which ProtParam (available online: http://web.expasy.org/protparam/) was used to determine the basic physical and chemical parameters of the proteins. The MEGA-X program (available online: http://www.megasoftware.net/) was used for a multiple sequence alignment. The phylogenetic relationships among the TLPs were determined according to the neighbor-joining method. The transmembrane regions were predicted, protein parameters were analyzed, signal peptides were predicted and three-dimensional structures were constructed, as described by Meng [33]. The three-dimensional structures were evaluated by the MolProbity program (available online: http://molprobity.biochem.duke.edu/) in order to check the correctness of the structure over the localized regions. The data, including the gene expression patterns, were expressed as the mean ± standard error of the mean. Statistical analyses were performed using SPSS 17.0 (SPSS Inc., Chicago, IL, USA), and a one-way analysis of variance with a multiple comparisons test (Duncan’s test) was used to determine the statistical significance between the treatments, with significance being defined as *p* < 0.05.

## Figures and Tables

**Table 1:** Amino acid composition of *Bx-tlp-2.*

Amino acids	Number	Percentage	Amino Acids	Number	Percentage	Amino Acids	Number	Percentage
Ala (A)	4	4.7%	Gly (G)	11	12.9%	Phe (F)	5	5.9%
Arg (R)	4	4.7%	His (H)	1	1.2%	Pro (P)	2	2.4%
Asn (N)	4	4.7%	Ile (I)	1	1.2%	Ser (S)	6	7.1%
Asp (D)	8	9.4%	Leu (L)	9	10.6%	Thr (T)	7	8.2%
Gln (Q)	1	1.2%	Lys (K)	1	1.2%	Tyr (Y)	7	8.2%
Glu (Q)	4	4.7%	Met (M)	2	2.4%	Val (V)	4	4.7%
Cys (C)	4	4.7%						

**Table 2:** Amino acid composition of *Bx-cpi*.

Amino acids	Number	Percentage	Amino Acids	Number	Percentage	Amino Acids	Number	Percentage
Ala (A)	10	8.1%	Gly (G)	4	3.2%	Phe (F)	7	5.6%
Arg (R)	4	3.2%	His (H)	7	5.6%	Pro (P)	5	4.0%
Asn (N)	2	1.6%	Ile (I)	5	4.0%	Ser (S)	4	3.2%
Asp (D)	3	2.4%	Leu (L)	13	10.5%	Thr (T)	3	2.4%
Gln (Q)	14	11.3%	Lys (K)	18	14.5%	Val (V)	22	17.7%
Glu (Q)	2	1.6%	Met (M)	1	0.8%			

## Supplementary Materials

Supplementary materials can be found at https://www.mdpi.com/1422-0067/21/3/982/s1.
