# Supplementary material for: Potential Molecular Mimicry Proteins Responsive to α-pinene in Bursaphelenchus xylophilus
Source: Int J Mol Sci. 2020 Feb 1;21(3):982. doi: 10.3390/ijms21030982 (PMC7037625; doi:10.3390/ijms21030982)
Supplement: Supplementary file 1 [file ijms-21-00982-s001.pdf]

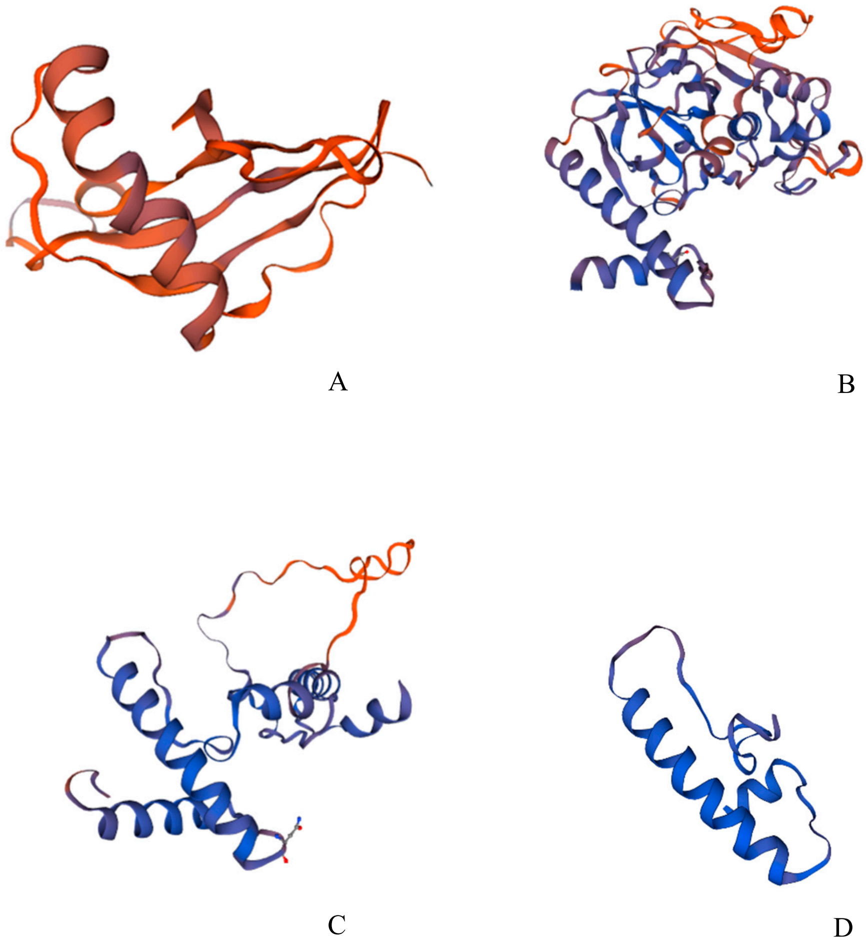

Figure S1. The picture of three-dimensional structure of Bx-CPI, Bx-CPL-1, Pm-CPL and cathepsin propeptide inhibitor domain (I29) (A, Bx-CPI; B, Bx-CPL-1; C, Pm-CPL; D, I29)

The inhibitor domain of Bx-CPL-1 contained in residue 86-137 amino acids, and that of Pm-CPL contained in residue 42-98 amino acids. The gene accession number of *Bx-cpi*, *Bx-cpl-1* and *Pm-cpl*: MK000288, EU651860 and AIF75887.

Table S1 The RPKM values of genes expressed in the comparative transcriptomics of *B. xylophilus*. RPKM, Reads Per Kilobase per Million mapped reads. AD, 56.33 mg/mL of α-pinene. AG, 214.5 mg/mL of α-pinene. CK, control.

Gene	Gene ID	Length	CK		AD		AG	
			Reads	RPKM	Reads	RPKM	Reads	RPKM
Bx-tlp-1	BUX.s00036.89	441	1989	413.4391	2588	418.1405	2175	430.9614
Bx-tlp-2	BUX.s00036.92	258	3	1.0659	10	2.7617	7	2.3708
Bx-cpi	BUX.s00351.347	375	1556	380.359	1286	244.3466	1301	303.1543
Bx-cpl-1	BUX.s01144.106	1203	2098	159.8657	2991	177.1525	2417	175.5613

Table S2 Real-time PCR primers sequences.

Gene	Primers	Sequence
Bx-tlp-1	TLP1-F	TGTGGCTGACACTTATGG
	TLP1-R	AGTCGTCGTTGTAGTTGATA
Bx-tlp-2	TLP2-F	TCACACTTGCCGAGTTCTCCTTC
	TLP2-R	TCCGTGAGTCTTGCTATTGTCTCC
Bx-cpi	CPI-F	CACGGCAAGTGCTAGGTGGATT
	CPI-R	TGAGCAGCGACAACTTGATGGAA
Bx-cpl-1	CPL1-F	GACTGGAGAGAAAAGGGCGTT
	CPL1-R	CCGTGAGCGATTGCGTAC
β-actin	NCF	TCCGTACCCTGAAGTTGGCTAACC
	NCR	AAGTGGAGACGAGGGAATGGAACC
Pm-tlp	Pmtlp-F	GCTGGTTGAATATACTCTGAAT
	Pmtlp-R	GGGAGATAGGGACATTGAA
Pm-cpi	Pmcpi-F	GATGGCTCTGCGTATTGT
	Pmcpi-R	CTTGGCTTCTATTGTAAGGTAG
actin	ACF	CATAGTTTTGTGTGCTCTGTT
	ACR	TGCGTCATCCCACTATTCAT
